# Exercise Selection and Common Injuries in Fitness Centers: A Systematic Integrative Review and Practical Recommendations

**DOI:** 10.3390/ijerph191912710

**Published:** 2022-10-05

**Authors:** Diego A. Bonilla, Luis A. Cardozo, Jorge M. Vélez-Gutiérrez, Adrián Arévalo-Rodríguez, Salvador Vargas-Molina, Jeffrey R. Stout, Richard B. Kreider, Jorge L. Petro

**Affiliations:** 1 Research Division, Dynamical Business & Science Society—DBSS International SAS, Bogotá 110311, Colombia; 2 Research Group in Physical Activity, Sports and Health Sciences—GICAFS, Universidad de Córdoba, Montería 230002, Colombia; 3 Research Group in Biochemistry and Molecular Biology, Faculty of Sciences and Education, Universidad Distrital Francisco José de Caldas, Bogotá 110311, Colombia; 4 Sport Genomics Research Group, Department of Genetics, Physical Anthropology and Animal Physiology, Faculty of Science and Technology, University of the Basque Country (UPV/EHU), 48940 Leioa, Spain; 5 Research and Measurement Group in Sports Training (IMED), Faculty of Health Sciences and Sports, Fundación Universitaria del Área Andina, Bogotá 111221, Colombia; 6 ARTHROS Centro de Fisioterapia y Ejercicio, Medellín 050012, Colombia; 7 Centro de Capacitación en Acondicionamiento y Preparación Física (CCAPF), Bogotá 111511, Colombia; 8 Faculty of Sport Sciences, EADE-University of Wales Trinity Saint David, 29018 Málaga, Spain; 9 Physiology of Work and Exercise Response (POWER) Laboratory, Institute of Exercise Physiology and Rehabilitation Science, University of Central Florida, Orlando, FL 32816, USA; 10 Exercise & Sport Nutrition Laboratory, Human Clinical Research Facility, Texas A&M University, College Station, TX 77843, USA

**Keywords:** resistance training, musculoskeletal pain, exercise movement techniques, muscle strength, sports injury

## Abstract

Weight resistance training (RT) is an essential component of physical conditioning programs to improve the quality of life and physical fitness in different ages and populations. This integrative review aimed to analyze the scientific evidence on the relationship between exercise selection and the appearance of musculoskeletal injuries in physical fitness centers (PFC). The PubMed or Medline, EMBASE or Science Direct, Google Scholar and PEDro databases were selected to examine the available literature using a Boolean algorithm with search terms. The review process was performed using the five-stage approach for an integrative review and it was reported according to the PRISMA in Exercise, Rehabilitation, Sport Medicine and Sports Science (PERSiST) guidelines. A total of 39 peer-reviewed articles (Price index = 71.7%) met the inclusion criteria and evaluated the link between exercise selection and the incidence of injuries in exercisers who regularly attend PFC. Most injuries occur to the shoulders, elbows, vertebrae of the spine, and knees. Although the injury etiologies are multifactorial, the findings of the reviewed articles include the impacts of overuse, short post-exercise recovery periods, poor conditioning in the exercised body areas, frequent use of heavy loads, improper technique in certain exercises, and the abuse of performance- and image-enhancing drugs. Practical recommendations addressed to clinical exercise physiologists, exercise professionals, and health professionals are given in this paper. The exercise selection in RT programs requires professional supervision and adhering to proper lifting techniques and training habits that consider the anatomical and biomechanical patterns of the musculoskeletal structures, as well as genetic, pedagogical, and methodological aspects directly related to the stimulus–response process to mitigate the occurrence of RT-related injuries in PFC.

## 1. Introduction

Due to the improvement in neuromuscular performance, weight resistance training (RT) is an essential component of physical conditioning programs that seek to improve activities of daily living, self-care, and the quality of life in different ages and populations [1,2]. In fact, the improvement in health-related variables is associated with the augmentation of muscle mass and strength levels [3]. In this sense, several authors consider muscular strength as a transversal axis within physical exercise programs [4]. Notably, the physiological adaptations generated by the strength training (i.e., maximal dynamic strength, local and global muscular power or endurance) benefit other physical abilities such as cardiovascular fitness, balance, range of motion, and speed both in untrained subjects and elite athletes [5,6,7,8]. Thus, an adequate prescription of weight RT, as a method of strength training, based on the individual response and objectives or adaptations to be achieved is extremely important.

Clinical exercise physiologists, exercise professionals [9], and athletic trainers [10] are in charge of designing physical exercise programs either for prescribing exercise, promoting regular physical activity, or reaching fitness or performance goals. To give an adequate dosage of exercise stress-induced stimuli, exercise professionals and personal trainers have to consider the individual characteristics (e.g., genetics, developmental conditions, morphological features demographics, environment, etc.) and the adaptive response [11,12]. Thus, the exercise dosage should be given within an orderly scheme of actions based on: (i) planning, where the establishment of periods (periodization) and more specifically the programming would indicate the number of days to train (frequency), according to the availability of the subject; (ii) a greater or lesser rest time between the exercise sessions (density); (iii) a necessary number of repetitions above or below the subject’s rate of perceived exertion that also considers the movement velocity during RT as a standardized method for load progression (intensity). In general, the whole scheme of actions encompasses a maximum number of repetitions per body region (methodological organization) that is divided into almost equal subunits of muscular stress (sets) with adequate rest (pauses) between them (volume) [13,14,15]. 

The aforementioned frames the operative elements of exercise prescription, understanding it as “the professional work consisting of prescribing, ordering, or determining a set of physical exercises aimed at maintaining or improving physical condition or health” [16]. It must be noted that postural malalignments associated or not to sedentary behavior [17], altered motor control [18], reduced physical fitness [19], and poor exercise execution [20] are among the potential risk factors for exercise-related injuries. Indeed, several cases of postural problems and morphological alterations in both the general [21] and young [22] populations are associated with musculoskeletal dysfunctions or physical injuries that in some cases have not been adequately treated by the health care institutions [23,24]. Hence, some concerns and questions that need to be answered with scientific argumentation beyond expertise and pragmatism arise regarding the exercise selection and the potential injury risk within physical fitness centers (PFC): When to include a given exercise? Does the exercise order matter? How do movement patterns impact on exercise outcomes? Is there a higher risk of injury when doing certain exercises? This represents a challenge for the exercise professionals and personal trainers at the moment of adequately selecting the exercises that will be prescribed in the training program. It can be assumed that prescribing exercise should consider aspects related to exercise dosage (magnitude of the stimulus), body posture, and biomechanics, especially in some body regions such as the craniocervical region and its impact on motor control, disturbances of anatomical pathways, and the myofascial network or meridians. Notwithstanding, there is no clarity on this in the current literature. Thus, the purpose of this integrative review was to analyze the scientific evidence on the relationship between the selection of weight resistance exercises in physical fitness programs and the potential appearance of musculoskeletal injuries within PFC in healthy individuals.

## 2. Methods

This study implemented the five-stage approach of Whittemore and Knafl [25], which is used as the established guideline for an integrative review. We aimed to synthesize the available literature regarding exercise selection and injuries reported in PFC through the combination of experimental and non-experimental studies, which allows for greater impacts when reporting evidence-based recommendations. Nevertheless, the basic methodology was optimized by improving the problem formulation, literature search, evaluation, analysis, and presentation of findings stages in order to systematize the review process and improve the scientific robustness according to the recommendations given by Hopia et al. (2016) [26] and the PRISMA in Exercise, Rehabilitation, Sport Medicine and Sports Science (PERSiST) guidelines [27]. Considering that this review was not eligible to be registered in PROSPERO, as it focused on physical fitness and performance, the summary information was uploaded to Figshare to make it publicly accessible in order to avoid unnecessary duplication (doi: 10.6084/m9.figshare.20412486).

### 2.1. Eligibility Criteria

The inclusion criteria for this systematic review were as follows: (1) experimental or theoretical articles (randomized controlled trials, case studies, narrative reviews, systematic reviews, and meta-analyses—the latter must comply either with PRISMA COCHRANE declarations or be registered in PROSPERO); (2) peer-reviewed articles published between 2009 and 2021; (3) publications written in English, Spanish, and German; (4) full text available; (5) articles focused on exercise selection and RT programs on different physiological or anatomical markers related to the development of injuries and epidemiological analysis of injuries in healthy subjects across the lifespan who attend athletic, private, public, or school or university PFC. Studies that did not correspond to original research (e.g., editorials, notes, dissertations), focused on injury rehabilitation, or included patients suffering from any disease were excluded.

### 2.2. Information Sources

The PubMed or MEDLINE and EMBASE or Science Direct databases were selected to examine the available literature. Further papers were sought by hand-searching in Google Scholar and the PEDro database.

### 2.3. Search Strategy

The search was developed using free language terms related to exercise selection and common injuries in subjects who strength train in fitness centers. The following Boolean algorithms were used to perform the search: PubMed/MEDLINE *“exercise selection” OR “resistance training” OR “bodybuilding” AND (“risk factors” OR “sports injuries”) NOT disease NOT patient* and *“exercise selection” OR “bodybuilding” AND “injuries”*; EMBASE/Science Direct *(“exercise selection” OR “resistance training” OR “bodybuilding”) AND (“risk factors” OR “sports injuries”) NOT disease NOT patient*.

### 2.4. Study Selection and Data Collection Process

Three of the authors independently searched the selected databases for articles (D.A.B., L.A.C. and J.L.P.). Those publications that met the inclusion criteria were selected to continue with the data analysis and synthesis phases, for which a table was designed to report the results and compare the main findings (citation or country, language, type of study, methodology, and conclusions). Discrepancies were identified and resolved through discussion (with a fourth author when necessary). As with previous review articles published by our research group [28,29], we calculated the Price’s index to measure the obsolescence of the literature as the ratio of the number of references published in the last five years to the total number of references. The study selection took place during December 2021 and March 2022, although an updated search was conducted in June 2022 prior to manuscript submission.

## 3. Results

**Figure 1:**
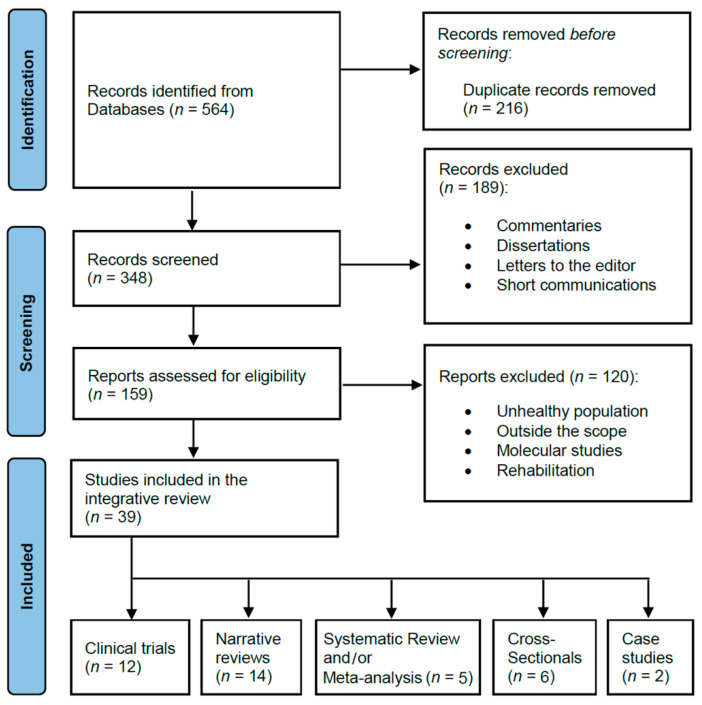
PRISMA flow diagram.

The preliminary search for abstracts, after using the Boolean search algorithm in the selected databases, generated 564 articles in total. After removing duplicates (*n* = 216), the abstracts were carefully reviewed (*n* = 348) for potential inclusion in the review; however, 309 papers were excluded after the evaluation process given they included populations with health problems, did not correspond to the types of articles detailed in the inclusion criteria, did not have a direct relationship with the proposed topics to be analyzed, were focused on injury rehabilitation, or did not have sufficient scientific robustness (published in predatory journals). Thus, a total of 39 peer-reviewed articles (Price index = 71.7%) met the requirements of this integrative review. Figure 1 shows the flow chart of the literature search.

The selected articles are synthesized in Table 1, in which the most relevant results are reported according to the aim of this integrative review. To minimize the risk of bias, the process of generating the table was supervised by at least two authors in order to discuss and compare the study conclusions until a consensus was reached.

The neuromuscular control and integrity of muscle and joint structures are compromised when some training principles are violated; therefore, it is very important to understand and integrate them into weight RT programs. DeWeese et al. [45] suggested developing exercise programs within periodization models, given that it is easier to manipulate training variables such as the intensity, volume, variation, and specificity of the exercises while avoiding overuse, allowing adequate overload and gradual increases to avoid abrupt stimuli that might increase the risk of injury. In fact, the RT program variables should be set according to the level of experience and learning capacity from the easiest to the most complex techniques [32,48]. Several articles have aimed to examine the impact of strength training (including weight RT) on reducing injuries in different anatomical locations while improving different parameters of physical fitness and body composition. The evidence shows injury-preventing structural and neural adaptations in the musculoskeletal tissue (e.g., increase in the tensile properties of hamstring muscles, augmented length in the long head of the biceps femoris, protective effects of eccentric actions on knee flexors) after weight RT provided the selected exercises obey the biomechanical pattern of the joints and muscles involved [54]. Indeed, Escamilla et al. [37] found that the load generated on the anterior cruciate ligament (ACL) increased or decreased according to the degree of flexion of the knee joint, whether or not the exercise included body weight and variations in exercise technique. For instance, heavy RT may contribute to the optimal remodeling process and adaptations for injury prevention by increasing the endomysium content of collagen XIV, macrophages, and tenascin-C in the MTJ region [47]. 

However, there is a wide variety of injuries caused by strength training within the PFC or training programs, most of the time caused when fitness practitioners do not have adequate professional supervision. In this regard, when a qualified professional advises and follows-up the RT program, the predisposing factors and risk of injury decrease [31,32]. Thus, the injuries that can be caused by inadequate strength training, depending on the type of exercise practiced or the type of strength sport practiced [34]. Epidemiologically, shoulder injuries constitute a large proportion of RT-related injuries [43,57,61,63,67], followed by elbow or knee injuries and lower back pain, mainly due to inadequate exercise selection, technique, and overuse [34,41,67]. The susceptibility of the shoulder complex to RT-related injuries is due in part to the stress placed on a joint that is anatomically non-load-bearing but assumes the role of an active joint with high-load repetitive lifting [42]. For example, impingement injuries are possible when the subacromial space is at its smallest, at 120° humeral elevation, 90° abduction, and 45° external rotation [65]. This position is reached during the pull-up motion (when supraspinatus is most active), especially with a narrow grip such as during front and reverse pull-ups (since it increases the required abduction moment). Nevertheless, wide pull-ups reduce the high stresses in both the deltoid and supraspinatus by emphasizing the back muscles (i.e., latissimus dorsi, trapezius, and rhomboid major) [65]. It is noteworthy that the injury risk might be higher when the fatigue level rises due to the increased eccentric loading on the supraspinatus. Similarly, the end-range “high-five” position (upper arm in 90° abduction, elbow in 90° flexion, and terminal external rotation of the shoulder) has been described as a posture of high injury risk during resistance exercises [33]. 

At the knee level, the analyzed articles indicated a moderate prevalence of injuries in this area caused by multiple factors [43,44]. For instance, patellofemoral pain is attributed to overuse, a lack of sufficient recovery periods, high loads without adequate prior conditioning, and an absence of technique supervision [30]. Although the prevalence of ACL rupture in PFC is low [57,62], the RT-related injuries can occur with movements of extreme internal rotation of the femur, with external rotation of the tibia, or when there is a large anterior displacement of the tibia [69]. Some factors have been found to increase or decrease the risk of injury in strength training. Jamison et al. [38] compared two RT programs, one of them with quasistatic trunk stabilization, and found a higher risk of ACL rupture in the program without trunk stabilization due to an increase in the tension of this ligament. Likewise, Lauersen et al. [56] concluded that improving strength and coordination in the knees, pelvis, and core might support the prevention of ACL injury and reduce anterior knee pain. Recently, Ferri-Caruana et al. [66] showed that an in-season 8-week pelvic and core strength training program reduced the values of ACL injury risk factors. It is important to note that the stress on the ACL is greater when lower-limb strength exercises do not support the body mass (double-leg and single-leg squats, lunges) [37].

On the other hand, despite their prohibition, fitness practitioners have shown a high prevalence in the use of pharmacological ergogenic aids [70], particularly the so-called performance- and image-enhancing drugs (PIEDs) [71]. These are considered doping substances and include synthetic derivatives of testosterone known as anabolic androgenic steroids (AAS), which have anabolic (related to the growth and increase in muscle mass) and androgenic (related to male sexual characteristics) effects [72]. The use of AAS is frequent in amateur and competitive bodybuilders [73], and importantly has been associated with an increased risk of musculoskeletal injuries [30,46,48,74], besides pathological issues derived from its injection, such as avascular lesions (necrosis) in the muscle and proteinaceous or hemorrhagic lesions in the intramuscular self-administration areas [75,76]. 

## 4. Discussion

This systematic integrative review aimed to summarize different aspects of exercise selection along with the incidence of injuries in exercisers who perform RT programs in PFC. The collective findings of this study indicate that the selection of exercises in weight RT programs and their relationship with the occurrence of injuries is multifactorial in nature (based on pedagogical, methodological, genetic, biomechanical, and anatomical–physiological principles). 

### 4.1. Underlying Factors

Similar to any other biological system, all principles converge to establish an optimal allostatic load that evokes adaptation and supercompensation (reach a new level above the initial value) [77]. It is worth noting that the allostatic load is defined as the cost that biological systems have to pay for being forced to adapt to a new set point [78]. Hence, the human body is able to reset the primary mediators of the physiological, anatomical, or psychological response at a new level that is different from the normal (homeostatic) operating range in a process that is called allostasis or “stability through change” [79]. Importantly, exercisers need to fulfill not only their energy, macronutrient, and micronutrient requirements but also their post-exercise recovery time requirements for adequate functioning in cases of exercise-induced physiological stress and subsequent adaptation [11]. For example, a relative energy deficiency in sports and suboptimal sleep may place athletes or exercisers at greater risk of musculoskeletal injury and bone stress fractures [80,81]. In agreement with Jagodzinski et al. [30], a similar discussion on this allostatic basis highlights a plethora of injury causes ranging from genetics to the individual’s medical history, training load, psychological factors (e.g., fear), and adaptive response.

**Figure 2:**
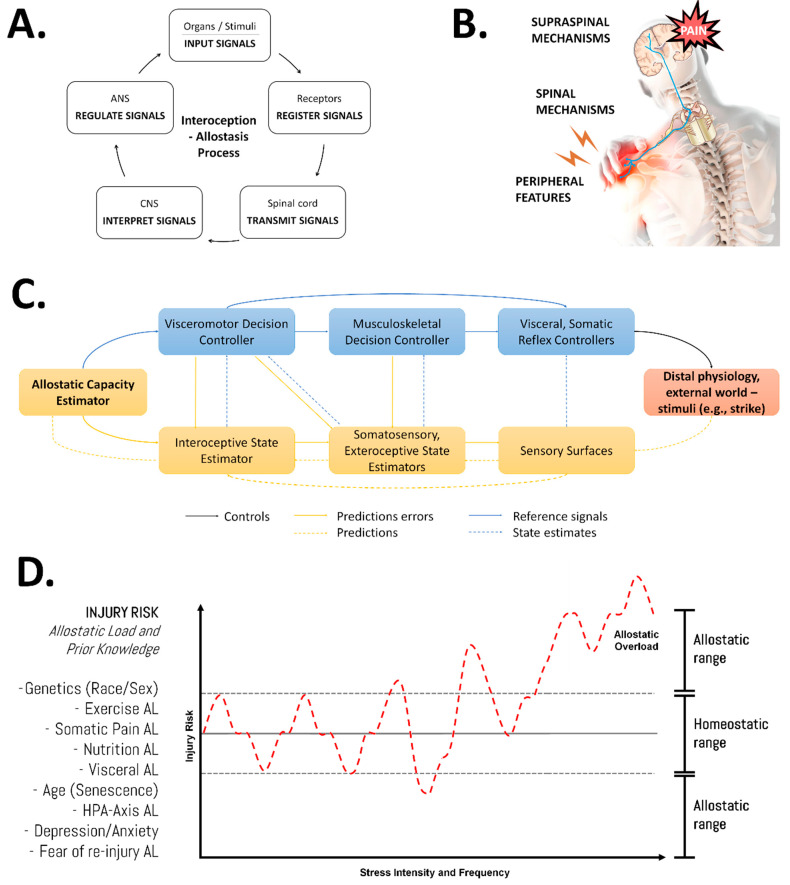
An integrative view of the multifactorial nature of injury risk. (**A**) General features of the interoception–allostasis process. While allostasis represents the adaptive process of stability through change, interoception refers to encoding representations of the internal (physiological) state of the body [86]. (**B**) Modulation of endogenous pain. Nociplastic pain conditions include the combination of central and peripheral pain sensitization, hyper-responsiveness to painful and non-painful sensory stimuli, and associated features (fatigue, sleep, and cognitive disturbances) [87]. (**C**) Detailed representation of the interoceptive–allostatic control (as a closed-loop system) of the human body in response to any stimuli. The injury or pain etiology might be discussed in terms of the role of the input signal (stimuli—distal physiology or external world), receptors (sensory surfaces, biological receptors), transmitters (spinal cord, anatomy trains), decoders (central nervous system), regulator elements (autonomic nervous system), and output signal (response, physiological effects). This block diagram was taken from Sennesh et al. (2022) [88]. (**D**) Representation of the changes in injury risk in response to stress exposition. Importantly, exercise selection is one of the many factors that might impact the allostatic load in fitness practitioners; therefore, it might influence the musculoskeletal or connective tissue overload. However, several other variables should be considered to avoid the risk of injury in PFC. AL: allostatic load. Source: designed by the authors (D.A.B.).

Clinical exercise physiologists, exercise professionals, and athletic or personal trainers should adopt a systemic (integrative and multifactorial), evolutionary (intuitive), and adaptive (ever-changing based on individualization) perspective or ‘bio-logic approach’ [82] that allows an understanding of the flow of information through interactions between system components and their regulatory aspects for a given phenotype and the allostatic load (Figure 2). In fact, the allostatic load has been proposed as a promising and underutilized measure that might be useful to assess the spinal cord injury time course [83]. This is in agreement with the fact that the total workload (with changes in external or internal loads) seems to affect injury prevention or management the most [84]. A multidomain intelligence platform that not only identifies target deficits that underlie second injury risk in sports (i.e., phenomic profiling) but also drives precision treatment to optimally enhance the athlete’s functional adaptability has been recently developed [85]. 

### 4.2. Exercise Modifications

As an element of the connective tissue, the anatomy trains cover most of the body and their relevance lies in the continuity between anatomical regions and the role in coordinating muscle activity, besides acting as a proprioceptive organ [89]. The anatomy trains might represent one of the mechanisms of transmitting, interpreting, and regulating external signals in the human body, including (i) the spiral line, (ii) the superficial back line, and (iii) the superficial front line. For instance, knee and shoulder pain or injuries have been associated with a compromise of the spiral line, which has helical paths that originate in the skull and then pass through the contralateral shoulder, serratus, anterior superior iliac spine, tensor fascia latae, lateral tibial condyle, and tibialis anterior, finally reaching the fibularis longus tendon [90,91]. However, it is important to point out that this theoretical model that aims to represent the human body through a fascial continuum requires further research due to the lack of scientific consensus [92].

**Figure 3:**
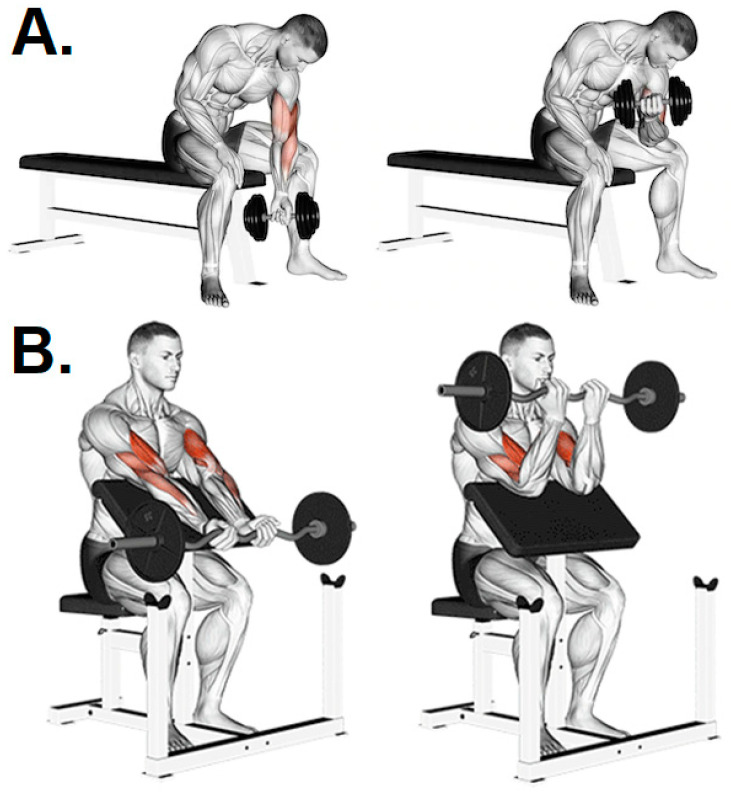
Seated bicep curls: (**A**) one-arm dumbbell concentration curl increases thoracic–lumbar kyphosis; (**B**) EZ bar Scott (preacher) curl provides proper positioning of the cervical and thoracic vertebrae. Source: Taken from Gym Visual available at https://gymvisual.com/ under copyright and owned by Aliaksandr Makatserchyk. Accessed on 14 January 2022.

Maintaining balanced posture is an aspect to be considered in the selection of exercises and their methodological progression. The degree of instability that could be achieved during certain RT movements requires greater activation of the stabilizing muscles of the trunk, which influences the rate of force development [93]. Therefore, performing any upper limb RT exercise unilaterally with free weights, a pulley cable, or an elastic band (standing, seated, or kneeling) will cause the increased resistance on one side of the body to significantly impact the stabilization demands at the central level, providing additional core muscle activation and stress [94]. Interestingly, two randomized controlled trials have shown that core strength training would not only reduce the ACL injury risk but also improve athletic performance [66,68]. However, it needs to be noted that some unstable positions or postural malalignments during strength exercises might put at risk the spine if high loads are used [95,96]. Furthermore, during typical activities of daily living, individuals frequently perform inadequate body positions or physical movements (with no mention of predisposing factors) that might cause spine alterations such as kyphosis, lordosis, and scoliosis [97,98,99], which can increase the risk of acute injury during weight RT programs. For example, some biceps exercises involve positions that increase thoracolumbar kyphosis (e.g., dumbbell concentration curl) instead of movements that allow the same elbow flexion with thoracolumbar and humerus stability (Figure 3). 

**Figure 4:**
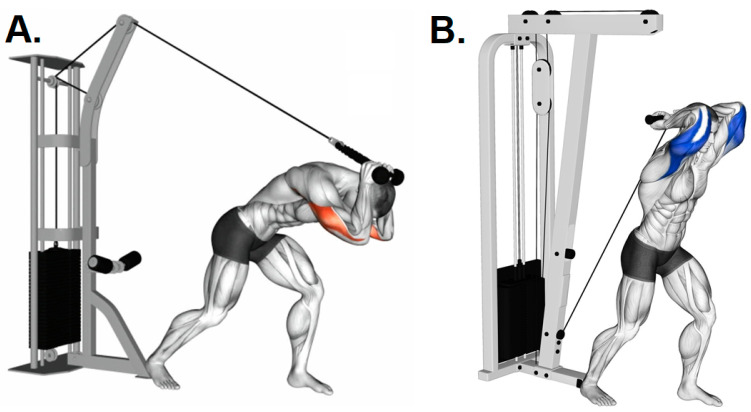
Standing cable overhead triceps extensions: (**A**) this posture generates tension in the myofascial and muscular chains in different ways; (**B**) recommended posture. Source: Taken from Gym Visual available at https://gymvisual.com/ under copyright and owned by Aliaksandr Makatserchyk. Accessed on 14 January 2022.

Another example of an exercise frequently used in the RT programs is the standing cable overhead triceps extension during the work of the triceps brachii. Inadequate use of the pulleys leads to a high level of stress on the lumbar spine when the trunk is brought forward and the flexed neck position. This generates a postural imbalance due to excessive tension of the myofascial and posteromedial muscle chains (Figure 4).

**Figure 5:**
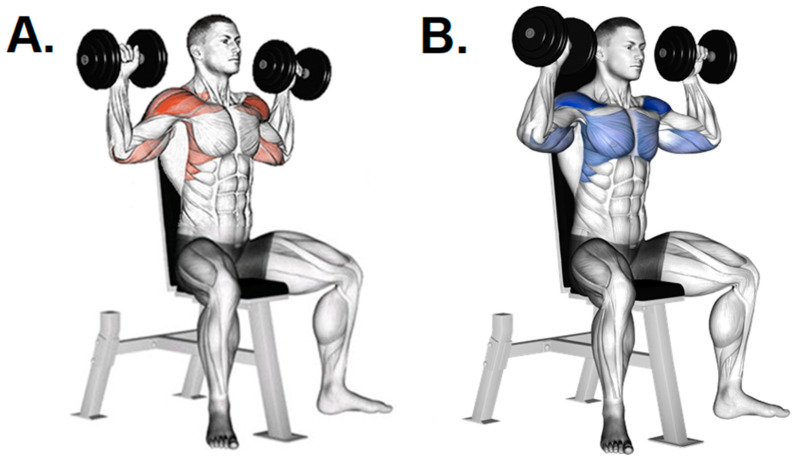
Representation of the “high-five” position: (**A**) traditional technique requiring a “high-five” end-position range (upper arm in 90° abduction, elbow in 90° flexion, and terminal external rotation of the shoulder), where the risk of injury increases with the anterior instability; (**B**) modification to avoid the “high-five” end-position. Raising the arms slightly in front of the head without extending completely in military press is advisable to decrease injury or pain risk. Source: taken from Gym Visual available at https://gymvisual.com/ under copyright and owned by Aliaksandr Makatserchyk. Accessed on 14 January 2022.

In ball-and-socket joints such as the shoulder complex, which was found to be one of the most common RT-related injury sites, the mechanical impact will depend on the movement of the humerus over other bone surfaces [100,101]. In fact, common weight RT exercises may place the shoulder in unfavorable positions that require the arm to extend horizontally behind the neck or may require the “high-five” position of being abducted and externally rotated (Figure 5).

Likewise, behind-the-neck presses lead to a dangerous position where the scapulohumeral portion reaches horizontal abduction along with forced external rotation. This greatly affects the scapulothoracic friction with an important rotational compromise of the scapula (depending on mobility) and a reduction in the existing space of the subdeltoid region impacting the supraspinatus tendon and the subacromial bursa, among others [41]. Thus, the combination of a high load, several repetitions, and fatigue in maintaining an unfavorable position can generate undue stress on the shoulder complex and higher injury risk. Similarly, special attention should be paid when altering the positions of the hands and feet for higher muscle hypertrophy in the deltoid muscles or the gastrocnemius and soleus muscles, respectively [102,103]. In the case of the pectoralis major, the evidence is inconclusive as to whether different angles (incline, flat, or decline) might be better for muscle development or activation [104,105,106]; however, the wide grip should be modified to avoid excessive horizontal abduction and the externally rotated position [107]. Considering the abovementioned examples, the professional in charge of prescribing and selecting the strength exercises must analyze each one of them, evaluate the possible risk of injury (benefit/cost ratio), make the necessary adjustments during progressions, and follow training guidelines [49,50].

It is important to consider that the fibrous connective tissues, which inform about the positional state and joint kinesthesia to the CNS by means of mechanoreceptors, might present neurophysiological changes after an injury. Afferent activity induced by peripheral injury has been shown to trigger a long-lasting increase in the excitability of spinal cord neurons, profoundly changing the gain (or receptive capacity) of the somatosensory system (allodynamic response) [108]. The afferent information would involve changes in the reception caused by the joint deafferentation [109]. Here, interoception provides performance metrics for visceromotor regulation as a feedback element for allostasis [88] (Figure 2). It is worth noting that injury-related changes also include disturbances in anatomical pathways as a function of the fascial network and its distal dysfunctional components in anatomical areas (such as the shoulder and knee) [110]. Motor coordination changes occur constantly in cases of chronic musculoskeletal pain, including lumbar pain [111]. To clarify, nociception does not necessarily mean pain. Due to noxious stimuli, such as a muscle or joint injury, nociceptors are activated and produce pain; however, if the stimulus is repetitive, like in chronic lumbar pain or osteoarthritis, a sensitivity to the nociceptive system can be developed and this would increase the response to non-harmful stimuli [112]. Considering chronic pain is a widespread problem around the world, pain neuroscience education (PNE) has been developed as an approach to pain treatment. It positively influences brain maps associated with fear or beliefs about exercise as a painful activity, which may diminish menaces and strengthen safety [113].

### 4.3. Practical Recommendations

1. Establish the objectives prior to the start of the weight RT program and opt for a non-linear (undulating) periodization model ordering the exercises to be implemented. Undulating periodization of RT has been shown to be more effective than a linear approach to increase strength and body composition in several populations [114,115,116,117].
2. Consider all training variables that directly impact the targeted phenotype, regardless of the work demand by large or small muscle groups. Refer to Rosa et al. (2022) for a recent meta-analysis on the comparison of the effectiveness of single versus multijoint resistance exercises on muscle hypertrophy [118].
3. Dedicate familiarization training sessions for teaching, observing, or correcting exercise techniques. This needs to be done independently of the experience level of the athletes or exercisers and the resistance to be used (machines, free weights, suspension).
4. The principle of safety in RT indicates that the exercises selected must preserve the integrity and health of the subjects. Start from easy to more complex exercises (from machines to free weights with low load) or those that require greater control (free weights with moderate-to-high loads or suspension RT).
5. To reduce the risk of injuries, ask for any pre-existing injuries or medical conditions, monitor for fatigue, and modify or eliminate suboptimal movement patterns or exercises entirely in persons not capable of performing them [60].
6. The exercises selected should consider all of the muscles and joints involved in the biomechanical movement, avoiding methodological errors such as prescribing the “bench press” for the exclusive development of the pectoralis. All muscles involved in each exercise should be analyzed for a correct prescription, thereby avoiding overuse injuries in certain body regions [41,58].
7. Precondition the muscle regions involved in sequential exercises (derived from weightlifting) with pulling or pushing exercises.
8. Prioritize movements that require large muscle groups at the beginning of the session. In this sense, fitness practitioners should perform the most demanding, challenging exercises early in their training sessions and should avoid tiredness, fatigue, technical errors, and excessive overload [43].
9. Although further research is warranted, practitioners should be aware of the role of myofascial fasciae and muscle chains involved in human movement to avoid incorrect body positions. Readers are encouraged to read a recent systematic review on exercise interventions to improve postural problems [119].
10. The exercises selected should obey the optimal range of motion of the joints involved, avoiding overloading the joint limits.
11. Due to the frequent reinforcement in RT of the imbalance of the external rotators and scapulothoracic muscles to the internal rotators at the shoulder, besides the stretching of the internal rotators, the incorporation of exercises to strengthen the lower trapezius, scapulothoracic muscles, and external rotators might mitigate common strength imbalances (anterior shoulder instability) and pain.
12. To prevent frequent RT-induced shoulder disorders the end-range “high-five” position (upper arm in 90° abduction, elbow in 90° flexion, and terminal external rotation of the shoulder) should be avoided [33,41]. Therefore, practitioners are encouraged to select exercises that require bringing the bar to the front of the torso (latissimus pull-downs or barbell presses to the front) instead of behind the neck.
13. In case of musculoskeletal discomfort when performing one of the exercises, opt for another exercise that meets the same objective (i.e., changing the prescription and technique of the exercises). The injury risk in some exercises is easily “mitigated” by a variation in execution. For example, biceps curls with an EZ bar instead of a straight bar; triceps presses with a 45° grip or rope; and no maximum stretch in exercises such as pullovers, flies, and dips.
14. Core training should be considered for ACL injury prevention. It has been demonstrated that it improves the extremity alignment in the frontal plane and muscle activation during sports-related tasks [68].
15. The rating of perceived exertion and pain scales have been recognized as valid markers of internal load [120] and pain thresholds [121]. These easy-to-apply methodologies help to accurately monitor intensity and to adjust the RT program [11], taking advantage of the interoception process for control within the allostasis model [88]. PNE might be used as an intervention strategy to reduce kynophobia (refer to Vélez-Gutiérrez et al. [122] for a recent short commentary on cortical changes).
16. Amateur and competitive bodybuilders should be aware that the consumption of AAS is a significant risk factor for several musculoskeletal disorders besides upper- and lower-limb injuries. Relevant predictors of the high frequency of PIED use include age, sex, educational level, and socioeconomic status [123,124], as well as psychological aspects associated with the constant dissatisfaction with body image of people attending PFC (e.g., bigorexia) [125].

## 5. Conclusions

The selection of exercises and their relation to the potential injury risk in the weight RT programs relate to multifactorial inputs that include anatomical and biomechanical patterns of the musculoskeletal structures, as well as genetic, pedagogical, and methodological aspects directly related to the stimulus–response process. The most prevalent injuries occur in the joints of the shoulder, knee, elbow, and vertebrae of the spine. Musculoskeletal pain and injury risk are mostly caused by overuse, short recovery periods between sessions, improper technique, poor conditioning in these body regions, and the frequent use of high loads. Special care should be taken when monitoring PIED users. Besides summarizing the individual characteristics of the selected studies and discussing them as a whole to contribute to the design and development of future research, this paper provides theoretical aspects based on a ‘bio-logic’ approach and practical recommendations addressed to clinical exercise physiologists, exercise professionals, and athletic or personal trainers in order to improve the selection of exercises and mitigate the occurrence of RT-related injuries in PFC. Nevertheless, it should be emphasized that the prevention of injuries during strength-based RT programs has been clinically addressed at length in the sports field and less from the perspective of fitness in PFC, which warrants further research. In any case, “no pain, no gain” should not be a training maxim, as highlighted by Ritsch (2020) [61]. The key to the prevention of injuries in recreational weightlifters and bodybuilders is having professional supervision and adhering to proper lifting techniques and training habits that might positively impact the allostatic load and exercise-induced adaptations.

## Figures and Tables

**Table 1:** Synthesis of the selected articles for the integrative review.

Type of Study	Participants	Aim	Instruments or Procedure	Main Findings/Conclusions	Reference
**Narrative Review/Qualitative Analysis**	30 references (includes articles from their year of publication until 2009)	To analyze the influential factors in the development of patellofemoral pain syndrome.	Literature search of epidemiological and clinical aspects. A retrospective, citation-based methodology was applied.	Besides genetic characteristics or some noxa, patellofemoral malalignment, high loads and overuse, uncontrolled exercises, and AAS represent risk factors for injuries. Individual training concepts with controlled exercises that reduce peak loads are desireable.	Jagodzinski et al., 2009 [30]
**Cross-sectional/Quantitative Analysis**	*n* = 413 (216M; 197F) Only 60 subjects participated in the laboratory analysis.	To assess the predisposing factors, signs, and symptoms usually associated with OVR or OVT in members of PFC.	Questionnaire of 41 questions to evaluate OVR/OVT + POMS questionnaire + hematological and biochemical (CK activity, cortisol, total T, free T) analyses	The absence of predisposing factors and signs/symptoms associated with OVR or OVT states was evidenced in fitness practitioners in Sao Paulo, Brazil. Thus, detecting people engaged in excessive exercise training needs to be confirmed.	Ackel-D’Elia et al., 2010 [31]
**Narrative Review/Qualitative Analysis**	91 references from which 27 were clinical trials (from their year of publication until 2010)	To review the current epidemiology of injury related to the safety and efficacy of RT among youth and to provide age-appropriate training recommendations for children and adolescents	Literature search with practical and applied aspects. A retrospective, citation-based methodology was applied.	Lack of supervision in strength training in the youth population is associated with an injury rate due to inadequate technical execution and load control. Musculoskeletal injuries during RT do not appear to be superior to other sports modalities and activities.	Faigenbaum & Myer, 2010 [32]
**Narrative Review/Qualitative Analysis**	82 references (includes articles from their year of publication until 2009).	To present an overview of the literature pertaining to shoulder injuries in the RT population and to elucidate the etiological risk factors hypothesized to be associated with the more common injuries.	Literature search in PUBMED, CINAHL, SPORTDiscus, and OVID databases using key words including resistance training, shoulder, bodybuilding, weightlifting, shoulder injury, and shoulder disorder.	Up to 36% of documented RT-related injuries and disorders occur at the shoulder complex. Deltoid to external rotators and upper to lower trapezius muscles should be strengthened to mitigate strength imbalances associated with RT. Train flexibility to increase internal rotation and avoid the end-range “high-five” position may prevent the development of anterior shoulder instability and pain.	Kolber et al., 2010 [33]
**Narrative Review/Qualitative Analysis**	38 references (includes articles from year of publication to 2010).	To introduce the history of strength training, explain the many different styles of strength training, and discuss common injuries specific to each style.	Literature search with practical and applied aspects. A retrospective, citation-based methodology was applied.	The most common acute non-urgent injuries are muscular strains and ligamentous sprains, while urgent injuries are fractures, dislocations, and tendon ruptures. Patellar dislocation and acute meniscal tears are rarely reported. Most injuries are related to accidents or overexertion although each style of strength training has its own principal injuries, both acute and chronic, related to the individual technique.	Lavallee & Balam, 2010 [34]
**Narrative Review/Qualitative Analysis**	68 references (includes articles from year of publication to 2011).	To review the possible role and effects of eccentric strength training for injury prevention and use this, together with injury biomechanics, as a basis to suggest an eccentric exercise classification criteria applicable to the track and field athletes.	Literature search with practical and applied aspects. A retrospective, citation-based methodology was applied.	Hip flexion or knee extension exercises that actively lengthen the hamstrings should be trained. Focus on the long head of the biceps femoris, proximal semitendinosus, adductor magnus, and semimembranosus, based on plyometric and eccentric exercises.	Malliaropoulos et al., 2012 [35]
**Non-randomized prospective controlled study/Quantitative Analysis**	A cohort of children aged 7 to 9 years: 446 boys and 362 girls in the intervention group (2675 person-years) and 807 boys and 780 girls in the control group (5661 person-years).	To evaluate whether an extended exercise intervention program (40 min per day of school physical education for 4 years including strength-based exercises) in children could produce long-term skeletal benefits without increasing the fracture incidence.	BMC (g) and bone width (cm) were assessed by DXA of the whole body, lumbar spine, femoral neck, and trochanter + fracture index recorded in the city hospital records.	A 4-year exercise program in children aged 7 to 9 years increased bone mass and size without affecting the fracture risk. In the girls, there was a gradually higher gain in femoral neck and trochanter BMCs and femoral neck width with each tertile of higher duration of physical activity. A similar pattern was found in the children, reaching significance in the lumbar spine BMC and femoral neck width.	Löfgren et al., 2012 [36]
**Narrative Review/Qualitative Analysis**	63 references (includes articles from their year of publication until 2012)	To discuss the biomechanical factors related to ACL loading during common weight-bearing and non-weight-bearing exercises and to guide clinicians regarding exercise selection and progression for patients with ACL pathology or reconstruction.	Literature search on tensile strength and strain energy density data of the ACL in different exercises.	It highlights that the load on the ACL is greater in exercises without supporting body mass (double-leg and single-leg squats, lunges); for example, squatting with a vertical trunk position, which decreases hamstrings activity and increases quadriceps activity, leading to higher ACL loading. Caution should be taken in exercises with high knee flexion angle or heel raise since they generate more ACL loading.	Escamilla et al., 2012 [37]
**RCT/Quantitative Analysis**	*n* = 37 healthy males (only 22 were included in the analysis) were divided into two 6-week training groups: RT and RT + TS	To investigate the effectiveness of a training program including focused TS exercises on improving lower extremity biomechanics, athletic performance, and trunk or core measures (control, endurance, and strength) compared with a program incorporating only RT.	ACL strain analyzed by biomechanical loading without anticipation with Vicon MX-F40 and Bertec 4060-10 systems + trunk control and core strength using a sudden force release test with an OMEGA DM-465 load cell system + core endurance using McGill protocols for lateral plank, pronated plank, and trunk flexor exercises + lower limb strength + sports performance analysis.	Despite the lack of differences between groups, which may have been due to inter-subject variability and sample size, RT generated greater risk of ACL rupture while the RT + TS program was able to limit potential negative effects. In addition, only the RT + TS group generated improvements in CORE strength and endurance.	Jamison et al., 2012 [38]
**Prospective Clinical Trial/Quantitative Analysis**	*n* = 24 volunteers: eight RT-trained individuals using AAS; eight RT-trained individuals who had never used AAS; and eight subjects without any history of regular RT or AAS abuse	To examine in vivo mechanical properties of patellar tendons subjected to long-term exposure to overloading and AAS abuse by comparing highly trained individuals using AAS to trained and untrained subjects without any history of AAS.	Supplementation and drugs report + maximal knee extension isometric torque using an isokinetic dynamometer (System 3, Biodex Medical Systems) + muscle thickness, pennation angle, fascicle length, CSA of the patellar tendon, and tendon mechanical and material properties using ultrasonography (10–25 MHz transducer MyLab25)	The CSA of the tendon was greater in RT-trained subjects who did not use AAS. The patellar tendon generated more stiffness, more stress, and greater tensile modulus in subjects using AAS, which may limit tendon safety probably due to alterations in collagen remodeling.	Seynnes et al., 2013 [39]
**Narrative Review/Qualitative Analysis**	39 references (includes articles from year of publication to 2013).	To discuss the PRT-related injuries and present an overview of documented shoulder injuries among older adults, presenting a brief review of its anatomy, and to discern the possible mechanisms of injury and risk factors.	Literature search in PubMed database using the following specific search terms: strength-training injuries, resistance-training injuries, sports injuries in the elderly, shoulder complex, shoulder injury, and shoulder disorder.	The combination of unfavorable positions, fatigue, and unproper technique during exercises, such as bench press, behind-the-neck pull-downs, military presses, and chest flies may lead to AI and rotator cuff injuries (especially in older population). Preventive exercise based on the lower trapezius and external rotators is advised to mitigate strength imbalances.	Sousa et al., 2013 [40]
**Cross-sectional/** **Quantitative Analysis**	*n* = 159 healthy male subjects (123 with weight RT training experience and 36 with no history of weight RT participation as control group)	(i) To determine if men who participate in weight RT present with clinical characteristics of shoulder hyperlaxity and AI. (ii) To determine if there is difference between the presence of these conditions among weight RT participants when compared with a control group. (iii) To investigate the association of exercise selection with clinical characteristics of AI.	A detailed questionnaire to document specific training patterns that includedfrequency, presence of pain, and exercise selection + load and shift test (to detect and quantify anterior glenohumeral joint instability) + apprehension test (to detect anterior glenohumeral joint instability) + relocation test (to diagnose anterior instability). All measurements were performed on the non-dominant arm.	The rates of pain and positive apprehension and relocation tests were significantly higher in trained subjects than in untrained subjects. The injury risk in the weight RT population might be reduced by changes in exercise selection and technique. For example, it is advisable to make modifications in exercises such as behind-the-neck pull-down or military press by bringing the bar down to the front chest (avoiding the high-five position). Also, incorporating reinforcement work in the external rotators might prevent the injury risk for anterior shoulder instability and pain.	Kolber et al., 2013 [41]
**Narrative Review/Qualitative Analysis**	49 references (includes articles from their year of publication until 2014).	To overview the frequency, type, and developmental mechanisms of shoulder injuries as a function of various sports.	Selective literature search in the PubMed database taking into account authors’ experience and research results as well as international and national recommendations.	Acute (e.g., traumatic dislocations, acromioclavicular joint dislocations, traumatic tendon ruptures, labral and cartilage defects, and fractures) and chronic (e.g., bursitis and tendinitis, secondary forms of impingement with rotator cuff and labral lesions) injuries are are particularly common in throwing and impact sports (e.g., tennis, golf, handball, and volleyball) but also in contact and extreme sports (e.g., judo, martial arts, bodybuilding, weightlifting, motocross, and downhill mountain biking). The type and frequency of injuries are strongly dependent on the risk and load profile of the individual.	Doyscher et al., 2014 [42]
**Retrospective cross-sectional study/Quantitative Analysis**	*n* = 71 (54M; 17F) German elite and competitive bodybuilders (33.9 ± 9.2 years).	To investigate rates of injury, pain during workouts or overusesyndromes, as well as the influence of particular intrinsic and external factors	Five-parts questionnaire: demographics and general information + workout-related data and pain symptoms + frequency and localization of previous injuries or musculoskeletal disorders + general health disorders + lifestyle, nutrition, and medical therapy	The squat and bench press exercises most frequently generate pain in lower and upper limbs during workouts, respectively. The highest injury rates were found in the shoulder, elbow, lumbar spine, and knee (>40 years-old athletes exhibiting higher injury rates). The injury rate in elite and competitive bodybuilders (0.24 injuries per 1000 h of bodybuilding) is lower compared to other weightlifting (e.g., powerlifting, strongman, or Olympic lifting).	Siewe et al., 2014 [43]
**Narrative Review/Qualitative Analysis**	83 references (includes articles from year of publication to 2011).	(i) To provide a review of the literature with respect to regionspecific tendon properties, in association with patellar tendinopathy; (ii) to outline the automated tracking method as used by recent studies for the determination of regionspecific mechanical properties to inspire future research; (iii) to discuss potential treatment strategies for the management of patellar tendinopathy.	Literature search in PubMed database using the following specific search terms: patellar tendinopathy, patellar tendinitis, jumper’s knee, patellar tendon, tendon injury, region specific tendon properties, mechanical properties, tendon strain, treatment.	Patellar tendinopathy is a common musculoskeletal disorder affecting a wide range of amateur and elite athletes, especially those that participate in jumping events. Tendinopathy appears to result in an increased tendon CSA concomitant with decreased stiffness. The use of eccentric exercise or heavy–slow strength training can optimize the prevention and recovery of patellar tendinopathy and pain reduction in athletes. In general, any activity that exposes a region of the tendon to the largest forces could be considered a potential risk factor.	Pearson & Hussain, 2014 [44]
**Narrative Review/Qualitative Analysis**	49 references (includes articles from their year of publication until 2015).	Planning proposals for strength–power training, allowing for logical integration and manipulation of training variables including exercise selection.	Literature search with practical and applied aspects. A retrospective, citation-based methodology was applied.	To maximize the transfer-of-training effect and reduce injuries in strength or power athletes, it is doubtful that single-joint exercises will have as much impact on performance as multijoint training exercises. Advanced athletes require greater variation in exercise selection, volume, and intensity of training compared to beginning athletes.	DeWeese et al., 2015 [45]
**Cross-sectional/** **Quantitative Analysis**	*n* = 142 male bodybuilders (35–55 years); 88 reported consuming AAS in the last two years while 54 did not have used AAS.	To provide more quantitative data on the association of AAS use with tendon rupture by assessing the history of tendon rupture in a large cohort of AAS users and comparison nonusers.	Medical history + history of surgical interventions + epidemiological instrument + anthropometric assessment + anti-doping and PIEDs analysis in urine and hair + medical evaluation (cardiovascular function).	AAS abusers have a higher tendon injury risk than non-AAS-using bodybuilders. In addition, upper body tendon ruptures were exclusively reported in the participants who consumed AAS.	Kanayama et al., 2015 [46]
**RCT/Quantitative Analysis**	*n* = 15 (11M; 4F) divided into three groups: control, acute training group, and training group for four weeks.	To describe the distribution of macrophages and matrix proteins in human MTJ and adjoining muscle fibers and to investigate the influence of heavy RT (quadriceps and hamstrings muscle groups) on this distribution.	Sample from the semitendinosus and gracilis tendons + immunohistochemical analysis of collagen types and macrophage density. Subjects were healthy except for an isolated ACL rupture and scheduled for reconstruction surgery; however, they were able to perform daily activities so could not be classified as inactive.	Quadriceps exercises included leg press and leg extensions. Hamstrings exercises included Nordic hamstring, lying leg curls, supine one-leg curls, and reverse hyperextensions. The 4-week heavy RT program resulted in more collagen XIV, macrophages, and Tenascin-C content in the endomysium, which may indicate an optimal remodeling process and adaptations for injury prevention in the MTJ region.	Jakobsen et al., 2017 [47]
**Case study/Quantitative Analysis**	An amateur 25-years old bodybuilder with 4 years of experience.	To describe the case of a young man who self-aministred PIEDs and suffered from pain and reduced mobility of the right elbow for several months.	Medical history (repeated hospital admissions) + biochemical analysis + ultrasound and NMR imaging of the subject’s right arm + X-ray of the left shoulder.	X-ray showed osteoarthritic changes at the glenohumeral junction and reverse Hill–Sachs defect, in addition to abnormal hepatomarkers. The causes are attributed to the excessive use of PIEDs and injected oils, lack of load control, and psychological problems.	Hameed et al., 2016 [48]
**Systematic Review** **/Qualitative Analysis**	67 references (includes articles from January 1995 to January 2014).	To summarize and update scientific knowledge on different topics and guidelines related to the prescription of strength training in young prepubertal and adolescent populations.	Literature search in PubMed, Scopus, SportDiscus, ScienceDirect, and Google Scholar databases using the following specific search terms: children, adolescents, youth, youth athletes, pediatric, strength training, resistance training, weight training, motor performance skill.	Causes of strength-training-related injuries in young population are due to misuse of equipment, excessive loads, faulty techniques, or lack of qualified supervision. Avoid or minimize exercises that involve excessive load or compression or shear stress to the spine. Qualified adult instruction, low coach/athlete ratio, frequent and quality feedback, and execution of new exercises without fatigue are recommended.	Peña et al., 2016 [49]
**RCT/Quantitative Analysis**	*n* = 88 (59F; 29M) Portuguese adolescents (15-17 years old) were randomly assigned to a control (*n* = 46) or experimental (*n* = 42) group.	(i) To evaluate the effects of a 32-weeks resistance and stretching training program applied in physical education classes on forward head posture and protracted shoulder. (ii) To evaluate in adolescents submitted to strength and stretching exercises the effects of a 16-week detraining period.	Strengthening and stretching exercises + posture alignment assessment (cervical and shoulder angle) with photogrammetric method using a software for postural analysis + ASES questionnaire for self-assessment of shoulder pain and function, as well as neck pain.	A 32-week posture corrective exercise program (strength and stretching), in addition to physical education classes, improves postural control with increases in cervical and shoulder angles in adolescents aged 15 to 17 years. In addition, when a four-month detraining period is given, the adaptations are not lost.	Ruivo et al., 2016 [50]
**RCT/** **Quantitative Analysis**	*n* = 33 (15F; 18 M) physically active participants were randomly assigned to hip flexor training (*n* = 16) or control (*n* = 17); only 26 completed the study and were included in the analysis.	To investigate the feasibility and effect of a novel simple hip flexor strength training program in healthy subjects, using elastic bands as external loading.	Maximal isomeric hip flexion strength in the dominant leg using a hand-held dynamometer (Powertrack II Commander, JTECH Medical) + delayed onset muscle soreness (pain) using numerical rating scale + rate of perceived exertion using the BorgCR10 scale	A 6-week hip flexor strength training program using elastic bands with isometric action on the dominant leg improved hip flexor muscle strength by 17%. These types of interventions may favor the prevention and treatment of acute long-term hip flexor injuries, such as acute rectus femoris injuries and longstanding iliopsoas-related pain and impingement.	Thorborg et al., 2016 [51]
**Systematic Review/Qualitative Analysis**	20 references (includes articles from their year of publication until 2015).	To systematically review the injury epidemiology of weight training sports using a list of injury epidemiology outcomes advocated by the IOC and to evaluate if demographic features influence the injury epidemiology.	Literature search in PubMed, SPORTDiscus, CINAHL, and Embase databases using Boolean algorithms containing key words such as wound, rupture, sprain, strain, and tear in weight training sports (weightlifting, powerlifting, bodybuilding, strongman, Highland Games, and CrossFit).	Mild (exercise execution required modification) to moderate (stopped performing the exercise) injuries were reported in the shoulders, knee, and lower back. The injury rate in weight sports (≈1–2 injuries per athlete per year and ≈2–4 injuries per 1000 h of training/competition exposure) was lower than those reported in most team sports, with Highland Games and strongman having the highest rates.	Keogh & Winwood, 2017 [52]
**Narrative Review/Qualitative Analysis**	134 references (includes articles from 1982 to 2016).	To synthesize and review the most recent literature related to young athlete development as it pertains to resistance training and physical literacy.	Literature search in PubMed and SPORTDiscus databases using: strength training OR resistance training AND children, strength training OR resistance training AND adolescents, strength training OR resistance training AND injury prevention, physical literacy, and young athlete development.	Weight RT training serves as injury prevention. A multifaceted RT program with skilled instruction can ensure that diversification of motor skill development occurs before the onset of puberty, preventing patellofemoral disorders, fractures, or ACL tears.	Zwolski et al., 2017 [53]
**Narrative Review/Qualitative Analysis**	110 references (includes articles from year of publication to May 2017).	To provide an evidence-based framework for the selection of hamstring strengthening exercises that reduce the rate of injury to this muscle group.	Literature search in Scopus and PubMed databases using three Boolean algorithms.	The benefits of strength training may be due to increased biceps femoris long head fascicle length, possibly a rightward shift in the angle of peak knee flexor torque, and improved eccentric knee flexor strength.	Bourne et al., 2018 [54]
**RCT/** **Quantitative Analysis**	21M (22.4 ± 2.2 years old with at least three years of strength training experience performing traditional and Romanian deadlifts).	To determine which deadlift technique is a better training protocol between the conventional and Romanian deadlifts as indicated by the greater demand in muscle activities and joint kinetics.	Fifty-nine markers were placed on anatomical landmarks for kinematic analysis using a six-camera VICON motion capture system + ground reaction force data using AMIT force plates + EMG analysis using the Desktop DTS, a three-channel wireless system.	The conventional deadlift resulted in significantly greater EMG activities ofthe rectus femoris and gluteus maximus than those of the Romanian deadlift. Additionally, convenitional deadlift produced greater knee and ankle net joint torques than the Romanian version. In conclusion, the conventional deadlift might be a better technique for training lower extremity muscles.	Lee et al., 2018 [55]
**Systematic Review with Meta-Analysis/Qualitative and Quantitative Analysis**	Six references (includes articles from year of publication 2017). A total of 7738 subjects aged from 12 to 40 years.	To systematically identify and analyze qualitatively and quantitatively RCTs of strength training-based sports injury prevention programs.	Literature search in PubMed, Embase, Web of Science, and SPORTDiscus databases for RCTs using keywords related to exercise programs, prevention, injury, and diagnoses.	Significant reduction in acute hamstring and ACL injuries by incorporating strengthening of the frequently injured muscles. To prevent ACL injury and reduce anterior knee pain, it is advisable to improve strength and coordination in the knees, pelvis, and core. Incorporate familiarization phase, recovery weeks, and individualized programs to reduce the risk of injury from overuse.	Lauersen et al., 2018 [56]
**Cross-sectional/** **Quantitative Analysis**	*n* = 104 (53F; 51M) subelite Swedish powerlifters	To investigate the prevalence, localization, and characterization of injuries among Swedish subelite classic powerlifters, with an emphasis on differences between men and women, and to investigate whether training and lifestyle factors are associated with an injury.	Web-based adapted questionnaire: the majority of questions required a dichotomous answer (yes/no), while the other questions offered several categorical answers or called for an open-ended response.	The lumbopelvic region, shoulder, and hip present more injuries in both sexes (70% reported a current injury, and 87% reported being injured during the past 12 months). Women more frequently experienced injuries in the neck and thoracic region than men. Incorporating rehabilitation exercises, emphasis on technique, warm-up, and flexibility are associated with significant improvements.	Strömbäck et al., 2018 [57]
**Narrative Review/Qualitative Analysis**	62 references (includes articles from year of publication to 2016).	To review the National Strength and Conditioning Association’s 2016 Position Statement on Long-Term Athletic Development.	Literature search in the PubMed database using the following specific search terms: youth sports injuries, early sports specialization, training and maturation, training versus developmental stage, and long-term athletic development.	Weight RT has been shown to reduce the risk of injury in youth population. Besides adequate recovery time, it is recommended that children and adolescents train strength two to three days per week (between 60 and 80% 1RM, 8 to 15 reps). Exercises that increase the genu varu/valgus, joint hypermobility, leg length discrepances, pelvic rotation, height, muscle tightness, large Q angle, and ratio of explosive-to-static strength might increase the risk of injuries.	Walters et al., 2018 [58]
**RCT/** **Quantitative Analysis**	67 New Zealand school boys (aged 12–14 years) were randomily assigned to either combined RT (n = 21), combined RT + weightlifting (n = 19), or control (n = 27)	To investigate how combined RT with or without weightlifting movements affect injury risk factors (jump landing kinematics and interlimb asymmetry) as well as resistance training skill.	Anthropometric measures + resistance training skills battery + tuck jump assessment + single-leg horizontal jump + modified star excursion balance test + isometric midthigh pull test	A 28-week combined RT program improved tuck jump scores more than combined RT + weightlifting and regular physical education curriculum. Practitioners can use a combination of traditional RT, plyometric, and weightlifting training to reduce injury risk factors associated with jump landings and improve resistance training skill competency.	Pichardo et al., 2019 [59]
**Prospective Clinical Trial/Quantitative Analysis**	*n* = 100 (80F; 20M) healthy adults (22 to 64 years)	To identify the injury rate during HIFT (e.g., “boot camp”-style classes, military training, or CrossFit).	Self-reported injury rate and injury location + self-reported training time + demographic data + record of exercise types and pain	The injury rate during HIFT was 9.0 injuries per 1000 training hours. No sex differences were found. The most common locations injured were knees and back, particularly with non-ballistic weightlifting (free weights), plyometrics, and calisthenics, as well as burpees and squats.	Batterson et al., 2020 [60]
**Narrative Review/Qualitative Analysis**	55 references (includes articles from year of publication to 2020).	To overview injuries and overload damage in weight RT.	Literature search with clinical and practical aspects. A retrospective, citation-based methodology was applied.	The shoulder, knee, and lower back are the most common locations for injuries. Exercises such as behind-the-neck presses, behind-the-neck lat pulls, biceps curls with the straight bar, triceps presses with the straight bar, good mornings, and exercises in maximum stretch should generally be avoided.	Ritsch 2020 [61]
**Systematic Review/Qualitative Analysis**	12 references from 2010 to 2018.	To analyze in detail the prevalence of injuries occurring in training based on the CrossFit^®^, cross-training, or HIFT modalities.	Literature search in PubMed, Web of Science, SPORTDiscus, and Scopus databases using Boolean algorithms containing CrossFit, extreme conditioning program, cross-training, HIFT (high-intensity functional training), and HIPT (high-intensity power training).	The shoulder joint is the anatomical area with the highest prevalence of injury in CrossFit^®^, cross-training, or HIFT methodologies (9/12 studies). The rate or ratio of injury depends on a wide variety of variables to consider (previous injuries, protocol used, presence of qualified coaches, etc.).	Barranco-Ruiz et al., 2020 [62]
**Retrospective cross-sectional study/Quantitative Analysis**	*n* = 213 (112M; 101F) from three Brazilian PFC.	To verify the anatomical sites with the highest occurrence of injuries and the number and possible risk factors for injuries in HIFT practitioners in the last six months.	Questionnaire as retrospective survey to evaluate injury rate.	In total, 38.50% of the participants had suffered some type of injury caused by the HIFT training routine, and about 70.7% experienced their first injury only after initiating training. The injury rate was 7.1 injuries for every 1000 h of training (higher risk in advanced practitioners), and the majority of the injuries affected the shoulder, lumbar area, knee, and wrist. Injury causes were incorrect execution techniques, recurrent efforts, and high loads.	Texeira et al., 2020 [63]
**Case study with literature review/Quantitative and Qualitative Analysis**	A 26-years old elite weightlifter with 4 years of experience.	To describe the case of a elite weightlifter who suffered from bilateral quadriceps muscle/tendon rupture. Also, to assess the risk factors, types of presentation, and management of such cases by revieweling literature.	Medical history + clinical examination + NMR imaging of both legs + literature search in PubMed using Boolean terms with keywords such as bilateral quadriceps muscle, quadriceps muscle, rupture, tear, lacerations, and sports.	The case of the elite weightlifter who ruptured both his quadriceps muscles/tendons during competition was retrospectively found to have a history of AAS use. The literature review revealed 11 cases of sports-related bilateral quadriceps tendon ruptures, five of which were weightlifters and two were in bodybuilders (5/7 had a history of AAS use, infuencing the extensor mechanism strength).	Dhillon et al., 2020 [64]
**Clinical trial/Qualitative Analysis**	*n* = 11 (26.8 ± 2.4 years) healthy male participants with > 3 years of RT experience	(i) To quantify, with musculoskeletal modeling, the loading of key upper limb and torso muscles during several pull-up variants. (ii) To examine the effect of different kinematic strategies on muscle recruitments. (iii) To highlight potential injury risks in concentric loading of vulnerable structures in these tasks.	Retro-reflective passive markers (21) were placed on anatomical landmarks of the thorax, clavicle, humerus, and forearm, and a scapula tracker incorporating three markers was placed along the scapular spine + kinematic data using a 9-camera optical motion system (200 Hz Vicon) + external kinetic data using a force platform (1000 Hz Kistler) + UK National Shoulder Model was used to simulate biomechanics of the clavicle, scapula, humerus, and forearm	There is potential injury risk in concentric loading of vulnerable structures, specifically the rotator cuff muscles, under complex and strenuous movement patterns involving high upper limb elevation. Given their heavy load and multiplanar complexity, pull-ups should be implemented as a late-stage component in shoulder rehabilitation and conditioning programs. All three pull-up variants (front, wide, and reverse grips) should be incorporated into the exercise program with systematic progression to provide greater global strengthening of the torso and upper limb musculature.	Urbanczyk et al., 2020 [65]
**RCT/** **Qualitative Analysis**	*n* = 29 female soccer players (16.4 ± 1.6 years) were randomly assigned to experimental (*n* = 18) or control (*n* = 11).	To evaluate the changes in biomechanical risk factors for an ACL injury after participation in a pelvic and core strength training program in female team players.	All measures were collected during bilateral and unilateral drop jumps. Knee frontal plane projection angle + hip, knee, and ankle peak flexion angles + jump height	An in-season 8-weeks pelvic and core strength training program (twice per week) resulted in improvements on ACL injury risk factors and vertical drop jump performance. Strengthening this body part might support injury prevention while increasing jumping performance.	Ferri-Caruana et al., 2020 [66]
**Systematic Review/Qualitative Analysis**	11 references (includes articles from from year of publication to January 2020).	To analyse the literature concerning powerlifting injuries, focusing on the injury rates, areas of injury, and biomechanical movement analysis.	Literature search in PubMed and Google Scholar databases using the search terms powerlifting and injury.	The injury rates in powerlifting were between 1.0 and 4.4 per 1000 h of training (lower than other strength sports). Most injuries were found in shoulders, lower back, elbows, and knees. Experienced lifters were more prone to overuse or chronic injuries, whereas novice lifters were more likely to experience acute injuries.	Dudagoitia et al., 2021 [67]
**RCT/** **Qualitative Analysis**	*n* = 48 recreationally active men (22.4 ± 2.6 years) were randomly assigned to experimental (*n* = 32) or control (*n* = 16).	To examine the effects of core muscle strengthening on lower extremity joint kinematics and muscle activation of selected trunk and lower extremity muscles during side-step cutting.	Kinematic data of the trunk, hip, knee, and ankle joints using a 3-dimensional motion capture system (Motion Analysis Corp) + electromyography analysis (LXM5308) + core stability test using prone and sideplank endurance tests	A 10-week core strength training program alters the motor control strategy and at-risk biomechanical parameters associated with an ACL injury during the cutting maneuver by reducing the knee valgus and hip adduction angles and increasing the vastus medialis/lateralis and hamstring/quadricep activation ratio.	Jeong et al., 2021 [68]

AAS: anabolic androgenic steroids; ACL: anterior cruciate ligament; AI: anterior instability; BMC: bone mineral content; CK: creatine kinase; CSA: cross-sectional area; DXA: dual-energy X-ray absorptiometry; EMG: electromyography; F: females; Free T: free testosterone; HIFT: high-intensity functional training; IOC: International Olympic Committee; M: males; MTJ: myotendinous junction; PFC: physical fitness centers; PIEDs: performance- and image-enhancing drugs; POMS: profile of mood states; PRT: long-term progressive training; OVR: overreaching; OVT: overtraining; NMR: nuclear magnetic resonance; RCT: randomized controlled trial; RT: resistance training; Total T: total testosterone; TS: quasistatic trunk stabilization.

## Data Availability

Not applicable.
